# Assessment of current occupational safety and health regulations and legislation in the Caribbean

**DOI:** 10.26633/RPSP.2017.26

**Published:** 2017-03-23

**Authors:** Muge Akpinar-Elci, MyNgoc Nguyen, Marvin Randall, Satesh Bidaisee, Omur Elci, Olaniyi Olayinka, Julieta Rodriguez Guzman

**Affiliations:** 1 Old Dominion University Center for Global Health, Norfolk Virginia United States of America Old Dominion University, Center for Global Health, Norfolk, Virginia, United States of America.; 2 St. George’s University Department of Public Health and Preventative Medicine St. George’s Grenada St. George’s University, Department of Public Health and Preventative Medicine, School of Medicine, St. George’s, Grenada.; 3 Pan American Health Organization Special Program of Sustainable Development and Health Equity Washington, D.C United States of America Pan American Health Organization, Special Program of Sustainable Development and Health Equity, Washington, D.C, United States of America.

**Keywords:** Safety, occupational health, regulations, laws, West Indies, Seguridad, salud laboral, reglamentos, leyes, Indias Occidentales

## Abstract

Neglecting occupational safety and health (OSH) can have adverse and even deadly consequences. While OSH is important in any nation, the issue is particularly concerning in developing countries, including ones in the Caribbean. The purpose of this study, which was carried out in 2012 and 2013, was to examine the reasons for an apparent fundamental lack of awareness of OSH in the Caribbean. We conducted a descriptive study, in which a questionnaire was administered, via telephone, to key policy-making representatives from six English-speaking Caribbean nations, in order to assess the current OSH environment in their countries. We also did a situational analysis of current OSH regulations and legislation within the six countries. We found that that some of the countries’ OSH laws are out of date or are limited to a certain type of industry. We also found that there is very little documentation on research on exposure to and risks from hazards and on psychological and reproductive health as related to OSH. It is recommended that these Caribbean countries both increase national OSH awareness and strengthen enforcement of OSH regulations. Additionally, further assistance and a more coordinated effort from intergovernmental bodies could help build and fortify OSH systems in the Caribbean.

## RESUMEN

Descuidar la seguridad y la salud de los trabajadores puede tener consecuencias adversas e, incluso, letales. Si bien la seguridad y la salud de los trabajadores son importantes en todos los países, revisten particular importancia en los países en desarrollo, especialmente en aquellos ubicados en el Caribe. El propósito de este estudio, que se llevó a cabo en los años 2012 y 2013, fue examinar las razones de la evidente falta fundamental de conciencia sobre esta cuestión en el Caribe. Realizamos un estudio descriptivo, en el cual administramos un cuestionario telefónico a representantes clave de la formulación de políticas de seis países de habla inglesa del Caribe con el fin de evaluar la situación actual de la seguridad y la salud de los trabajadores en su país. También hicimos un análisis de la situación de los reglamentos y la legislación vigentes en los seis países. Observamos que las leyes de algunos países son obsoletas o están limitadas a un determinado tipo de industria. También observamos que es muy escasa la documentación sobre las investigaciones realizadas tanto acerca de la exposición a los riesgos como de la salud psicológica y reproductiva vinculadas a la seguridad y la salud de los trabajadores. Se recomienda que estos países del Caribe aumenten la concientización nacional sobre esta cuestión y fortalezcan el cumplimiento de los reglamentos pertinentes. Por otra parte, una mayor asistencia y un esfuerzo más coordinado de los cuerpos intergubernamentales podrían ayudar a establecer y fortificar los sistemas de seguridad y salud de los trabajadores en los países del Caribe.

There is a direct link between occupational safety and health (OSH) issues and national security, the local economy, the environment, and overall health (1, 2). Since most employees spend one-third or more of each workday on the job, OSH policies and laws play an important preventative role. However, OSH is often neglected, resulting in adverse consequences (3-7). In 2005, the International Labor Organization (ILO) estimated that worldwide 6 000 people die daily from work-related incidents, totaling over 2.2 million deaths annually. Additionally, occupational accidents and illnesses and the related absenteeism reduce the world’s total gross domestic product by 4% annually (5, 6).

Unfortunately, the exact causes behind the neglect of OSH policies and laws remains largely unknown in developing countries, including in the Caribbean. There are an estimated 283 million workers in total in the countries of Latin America and the Caribbean (LAC). Occupational fatalities in LAC nations are significantly higher than in the United States of America, at 0.135 and 0.005 per 1 000 workers, respectively. Despite this high toll in the LAC nations, OSH issues receive little or no attention (1, 3). Additionally, in the Caribbean, there is nearly no documentation on research on exposure to and risks from occupational hazards.

Therefore, in order to understand the reasons behind the lack of knowledge and the practice of OSH standards in the Caribbean, a questionnaire was given to key policy-making representatives in six Caribbean countries in 2012, and a situational analysis of current OSH regulations and legislation in the Caribbean was conducted in 2012 and 2013. The main objective of this study was to examine the inherent lack of awareness of OSH within the six countries.

## MATERIALS AND METHODS

We carried out a descriptive study in which telephone interviews were conducted with key policy-making government representatives from six English-speaking Caribbean countries in order to gain a better understanding of the current OSH environment of those nations. The countries were: from the south, Grenada and Trinidad and Tobago; from the north, Jamaica and the Bahamas; and from the central region, Saint Lucia and Barbados.

The questionnaire utilized in the telephone interviews was developed and validated by a nine-person expert panel consisting of representatives from the Pan American Health Organization (PAHO), as well as Caribbean academicians, health professionals, and politicians. These experts were recruited from professional networking meetings in the Caribbean and selected specifically because they are recognized experts within the field of occupational health and safety within the Caribbean. A survey was chosen instead of in-depth interviews because we wanted to gather more standardized data in order to make comparisons between countries.

We also conducted a situational analysis of current OSH regulations and legislation within the six Caribbean countries in order to assess safety and health conditions, employer responsibilities, OSH governing bodies, and compliance and enforcement of laws. Those OSH regulations and legislation were collected from the CARIBLEX database of the International Labor Organization (ILO) and from the Caribbean countries’ official government websites. We also investigated which of the countries had ratified the ILO’s C155 - Occupational Safety and Health Convention, 1981 (No. 155), which provides a framework for a safety and health culture at work (5). (C155 entered into force on 11 August 1983 and had been ratified by 66 countries around the world by the end of 2016).

Finally, we also recommend policies for improving the current awareness of, enforcement of, and compliance with OSH legislation within the six Caribbean countries.

Because of the nature of the methodology we used, the research study did not require evaluation by an institutional review board.

## RESULTS

Findings from the situational assessment of OSH regulations and legislation in the Caribbean (8-29) are given in Table 1.

Of the six countries, only Trinidad and Tobago had OSH regulations that were up to date, that is, had been updated within the preceding five years. Additionally, of the six countries examined, only Grenada had ratified C155. The Bahamas, Saint Lucia, and Trinidad and Tobago had regulations and legislation that touched upon minimum wages and equal opportunities for employees. The Bahamas, Grenada, and Trinidad and Tobago had regulations and legislation on labor relations or industrial relations.

The specific OSH questions and the answers from the administered questionnaire are presented in Table 2. All respondents to the questionnaire were key labor-office representatives who had responsibility for conducting inspections, making policy recommendations, and/or ensuring enforcement of OSH laws and guidelines. We interviewed one officer per country, except for Grenada and Saint Lucia, where two officers were interviewed in order to compile all the necessary information in our survey.

The major industries with the most reported OSH incidents or breaches were construction, agriculture, and tourism.

Employees and/or employers reported OSH issues to government safety officers in half of the countries.

In regards to research conducted on exposure and risk hazards, Barbados, Jamaica, and Trinidad and Tobago reported a lack of research, and the Bahamas, Grenada, and Saint Lucia conducted no research on exposure to risks and hazards.

Three of the countries reported that they did not collect baseline data to determine the risk that workers face from hazards. The other three nations stated they either lacked resources or had a weak legislative framework, which reduced the effectiveness of the OSH laws.

All six countries suggested that making OSH regulations more effective is critical for improving working conditions.

**TABLE 1.** Occupational safety and health regulations and laws in six Caribbean countries, as of 2012–2013

	Jamaica	The Bahamas	Barbados	Saint Lucia	Trinidad and Tobago	Grenada
Main OSH legislation	The Factories Act of 1961 (8)	The Employment Act of 2001 (12)	The Factories Act of 1986 (16)	The Employee (Occupational Health and Safety) Act of 1985 (18)	The Occupational Health and Safety Act of 2004 (21)	The Employment Act of 1999 (28)
Additional OSH regulation and legislation	Building Operations and Works of Engineering Construction (9) The Docks safety, health and welfare regulations (10) Green paper national workplace policy on HIV/AIDS (11)	The Minimum Wage Act of 2002 (13) The Industrial Relations Act of 2002 (14) The Health and Safety at Work Act of 2002 (15)	The Accidents and Occupational Diseases (Notification) Act (17)	Contracts of Service Act (19) Equality of Opportunity and Treatment in Employment and Occupation Act (20)	Equal Opportunity Act (22) Industrial Relations Act (23) The Maternity Protection Act of 1998 (24) Minimum Wages Act (25) Retrenchment and Severance Benefits Act (26) Trade Unions Act (27)	Labor Relations Act, 1999 (Amended in 2000 and 2003) (29)
Ratified C155^a^	No	No	No	No	No	Yes (July 2012)

***Source:*** Created by the authors, using results from the study.

a C155 = International Labor Organization’s C155 - Occupational Safety and Health Convention, 1981 (No. 155), which provides a framework that supports a safety and health culture at work.

**TABLE 2.** Responses to questionnaire on occupational safety and health from government representatives in six Caribbean countries, 2012

Question	Jamaica (Director of OSH, Ministry of Labour)	The Bahamas (Deputy Director of Labour)	Barbados (Chief Labour Officer of Ministry of Labour)	Saint Lucia (Chief Labour Inspector and one of the labour inspectors)	Trinidad and Tobago (Chief Labour Officer)	Grenada (Senior labour officer, Ministry of Labour (MoL); Senior Environmental Officer, Ministry of Health MoH)
Q1 – What are your responsibilities and administrative role concerning OSH?	Labor inspector	Supervise officers and inspectors	Labor inspector	Labor inspector: identify hazards and make recommendations to avoid possible OSH breaches	Ensure enforcement of OSH laws and regulations; investigate accidents, fatalities, and OSH breaches	MoL: investigate OSH breaches in the workplace MoH: conduct inspections and investigations in hotels, restaurants, and general environment
Q2 – Which ministries play a role in framing, enforcing, and ensuring compliance with OSH laws?	Ministry of Labour (primary role in framing and enforcing); Ministry of Health	Ministry of Labour; Ministry of Health; Department of Environmental Services, Ministry of Housing and Environment	Ministry of Labour (leader); Ministry of Health; Ministry of the Environment	Ministry of Education, Human Resource Development and Labour; Ministry of Health	Ministry of Labour; Ministry of Health; Environmental Management Authority (risk assessment)	Ministry of Labour; Ministry of Health
Q3 – What are the major industries?	Construction, agriculture	Tourism, oil, construction, agriculture	Construction, tourism, agriculture	Construction	Oil and gas, manufacturing, tourism	Agriculture
Q4 – What are the most reported OSH incidents or breaches?	Construction: falls and failure to use personal protective equipment Agriculture: injuries and fatal accidents	Tourism: slips and falls Construction: injuries and fatal accidents	Tourism: slips and falls	Construction: injuries and fatal accidents	Mostly accidents related to heavy machinery; ergonomic problems	Agriculture: injuries and fatal accidents Sick building syndrome
Q5 – What reporting mechanism is instituted for employees and employers in regards to OSH issues?	Lack of reporting	Informal relationship between inspectorates and workplaces	Employers report workplace OSH incidents	Direct (employee/employer) reporting to government safety officers	Accident registry; direct (employee/employer) reporting to government safety officers	National Insurance Scheme
Q6 – What kind of research is conducted in regards to exposure and risks to hazards?	Lack of research (insufficient staffing and resources)	None	Lack of research (insufficient staffing and resources)	None	Lack of research (responsibility of companies)	None
Q7 – What kind of baseline data is used to determine risk of workers to hazards?	Limited (insufficient staffing and resources)	Not collected	Need to create an OSH surveillance data system and use this data for informing legislators	Need to create an OSH surveillance data system	Not collected	Not collected
Q8 – What are the key determinants that compromise the effectiveness of OSH laws?	Legislative framework	Education and resources	Limited powers of OSH department	Education and awareness	Human resources	Legislative framework
Q9 – How can the enforcement of laws be improved?	Make regulations more affirmative and less like recommendations	Make regulations more affirmative	More substantial fines for employers Improve enforcement	Improve inspection systems to detect OSH breaches in individual sectors Workshops for labor officers, sponsored by the International Labor Organization	More prohibitive laws and improved enforcement of legislation Make OSH regulations more attractive to employers Better coordination between ministries	Better coordination between ministries
Q10 – What policy changes are necessary to make OSH laws more effective?	Make OSH legislation more affirmative and less like recommendations Create a better administrative framework Enforce laws better	Improve the current OSH laws Increase the authority of inspectorates	Increase the power of the OSH departments	Increase awareness of OSH laws Improve reporting of breaches.	Create a better OSH system to eliminate duplication of duties in ministries	Improve legislative framework for OSH
Q11 – What revisions do you think would make OSH legislation better?	Create legislation that facilitates more voluntary participation by employers	Make major revisions to current laws.	Make OSH legislation less vague in regards to reporting and defining OSH breaches	Need to review and update OSH regulations and legislation	Restructure and update current legislation to eliminate loopholes Increase the coverage of OSH acts Include major stakeholders in preparing OSH legislation	Need to review and update current OSH policies and acts

***Source:*** Created by the authors, using results from the study.

In addition, all six of the countries believe it is necessary to review and make major revisions to their OSH laws so that the laws can facilitate more participation by employers, improve defining and reporting of OSH breaches, increase worker protection, and eliminate loopholes.

## DISCUSSION

Most of the reviewed OSH regulations and legislation lacked clarity in defining OSH hazards. In addition, there was a lack of specific regulations, baseline data, and exposure limits for workplace hazards. Furthermore, except in Trinidad and Tobago, there was no research on workplace exposures and risk factors available to inform and guide legislators. There was more of a focus on physical hazards, and little on issues such as psychological stress or reproductive health. The limited number of OSH practitioners and weak governmental commitment to OSH may have contributed to these situations in the Caribbean.

The regulations and legislation also covered a limited number of industries, such as construction and agriculture. In some cases the regulations and legislation were outdated and ineffective in addressing current OSH issues. For example, the Jamaica Factories Act of 1961 lists women under the sections of the document concerning underage workers, and the act goes no further in addressing the many issues that women face in the workplace.

Our findings have specific implications for the safety and health of the labor force of the six countries assessed, which are discussed in the following three subsections.

### Passing new legislation and ratifying ILO conventions on OSH

For a number of years, ILO and the Caribbean Community (CARICOM) have been working on integrating labor law in the Caribbean region and drafting a model labor law for occupational safety and health and the working environment (30). It is imperative to update OSH laws based on the CARICOM model so as to eliminate old and ineffective legislation in the Caribbean region. Future OSH laws and policies should include provisions dealing with the informal sector, from which 48% of economic activity in the LAC countries originated in 1998 (1).

OSH principles are highlighted in various ILO conventions and in recommendations adopted by the International Labour Conference. Besides C155, also particularly important is C161 - Occupational Health Services Convention, 1985 (No. 161) (31). Ratifying OSH-related conventions such as C155 ensures that all stakeholders necessary for securing OSH protections are contributing to that effort (32).

Except for Grenada, which already ratified C155, the interviewees highlighted the importance of approving C155. However, no legitimate reason was given for not doing so. Policymakers may be reluctant to ratify such conventions because ratification might negatively affect international trade and labor relations (1). However, these countries should consider ratifying ILO conventions and enforcing them in order to have better OSH standards.

### Better OSH administration and improved coordination

These six Caribbean countries need to strengthen OSH standards by instituting a better OSH framework, creating more effective regulations and laws, and coordinating more closely among ministries. These systems should also ensure that all government agencies are sharing information on OSH concerns. Improved communication on OSH issues among all governmental agencies is critical for developing better OSH administration systems.

In addition, more effective collaboration with international agencies is needed (1, 33). The ILO’s affiliation with the ministries of labor and PAHO’s affiliation with the ministries of health can make a difference in strategies for advancing OSH in the region. A more coordinated approach by these and other agencies in assisting target countries is needed to change the culture and awareness of OSH.

### Additional training and education on OSH

More training and education (34) is essential to changing the OSH situation in these six countries. Government officers need to be trained to identify hazards and also understand the nuances of the industries that they investigate. Additionally, there is a need for an educated governmental and nongovernmental OSH workforce, such as safety, environmental health, and public health officers, along with health care providers, to research and identify OSH hazards, exposures, injuries, and diseases. These efforts could improve both OSH surveillance and reporting systems (1). Educating employers and employees about the many dangers of the workplace is also important.

Some interviewees said that there are current efforts to educate the general public about OSH laws and guidelines by means of seminars and other forms of information dissemination. Such efforts can provide a basic understanding of laws, personal rights, and ways of preventing OSH risks, while also encouraging the reporting of incidents.

### Limitations and strengths of this study

We only interviewed officials in the six Caribbean English-speaking countries, so the findings are not generalizable to the entire Caribbean region. The findings are also subject to information bias. For instance, the accuracy of information provided by informants depends on their knowledge and interpretation of OSH standards.

The authors attempted to minimize such bias by selecting informants who are experts in the OSH field. Additionally, the authors independently reviewed relevant OSH regulations and legislation for the six countries.

The findings reflect the state of OSH regulations and legislation at the time this study was conducted. In retrospect, an investigation more tailored to the individual countries might produce a better response and understanding of the major challenges in the region. However, enough information was obtained from the questionnaire and situational assessment to gain a better grasp of the OSH regulations and legislation of each of the six English-speaking Caribbean countries.

### Conclusions

To our knowledge, this study is the first to investigate the seeming lack of OSH regulations and legislation in the Caribbean. The interviews added knowledge and gave a broad outlook on the OSH issues in the six Caribbean countries. In general, these nations have a complex OSH regulation problem that requires a holistic approach. The primary area of concern in regards to OSH regulations and legislation was inefficiency at the national level. Many legislators of the six countries were still unaware of the importance of OSH issues, and that OSH extends far beyond protecting employees in the workplace. In reality, OSH is a matter of national security, as OSH issues may affect the environment, the economy, and the overall health and well-being of the general population. Legislators and policymakers have to realize the importance of OSH and develop strong, effective legislation and regulations in order to produce sustainable solutions. It is essential that these countries tailor the legislation based on the unique aspects of their own needs and priorities.

### Acknowledgments.

The authors thank all the persons who played a role in this research.

### Funding.

None.

### Disclaimer.

Authors hold sole responsibility for the views expressed in the manuscript, which may not necessarily reflect the opinion or policy of the *RPSP/PAJPH* or PAHO.
